# Free Versus Fixed-Ratio Combination of Basal Insulin and GLP-1 Receptor Agonists in Type 2 Diabetes Uncontrolled With GLP-1 Receptor Agonists: A Systematic Review and Indirect Treatment Comparison

**DOI:** 10.3389/fendo.2022.870722

**Published:** 2022-05-20

**Authors:** Han Na Jung, Yun Kyung Cho, Se Hee Min, Hwi Seung Kim, Ye-Jee Kim, Joong-Yeol Park, Woo Je Lee, Chang Hee Jung

**Affiliations:** ^1^ Department of Internal Medicine, Asan Medical Center, University of Ulsan College of Medicine, Seoul, South Korea; ^2^ Asan Diabetes Center, Asan Medical Center, Seoul, South Korea; ^3^ Department of Internal Medicine, Hallym University Sacred Heart Hospital, Hallym University College of Medicine, Anyang-si, South Korea; ^4^ Department of Clinical Epidemiology and Biostatistics, Asan Medical Center, University of Ulsan College of Medicine, Seoul, South Korea

**Keywords:** basal insulin, fixed-ratio combination, free up-titration, GLP-1RA, meta-analysis

## Abstract

**Introduction:**

This study evaluates the efficacy and safety of the free up-titration of basal insulin and fixed-ratio combination (FRC) of basal insulin and glucagon-like peptide-1 receptor agonists (GLP-1RAs) in type 2 diabetes mellitus (T2DM) patients inadequately controlled with GLP-1RA.

**Methods:**

With the use of a systematic literature review of PubMed, Embase, Web of Science, and the Cochrane Library databases through July 2021, randomized controlled trials that compared the free up-titration or FRC with remaining on GLP-1RA in T2DM patients uncontrolled with GLP-1RA were included. A comparison of adding basal insulin to maintaining GLP-1RA and an indirect comparison between the two strategies were conducted on the change in HbA1c, fasting plasma glucose (FPG), target achievement [HbA1c < 7.0%], and the risk of confirmed hypoglycemia. The Cochrane Collaboration’s tool was used to assess the risk of bias.

**Results:**

Two free up-titration and two FRC trials involving 1,612 participants, all lasting 26 weeks, were included. Both approaches significantly lowered HbA1c levels (weighted mean difference [WMD] −0.75%, 95% CI −0.97 to −0.53) but increased hypoglycemic risk [risk ratio (RR) 7.59, 95% CI 3.35−17.17] compared to the unchanged GLP-1RA. No significant differences were discovered between the two methods regarding the decrease in HbA1c (WMD 0.08%, 95% CI −1.07% to 1.23%), FPG (WMD −2.29 mg/dl, 95% CI −45.07 to 40.49 mg/dl), target achievement (RR 1.03, 95% CI 0.50−2.14), and hypoglycemic risk (RR 0.32, 95% CI 0.03−3.59).

**Conclusion:**

In patients who failed to reach target HbA1c levels despite the GLP-1RA treatment, both strategies of adding basal insulin, free up-titration and FRC, are comparable options are comparable options.

## Introduction

The era of having insulin as the only available injectable therapy in patients with type 2 diabetes mellitus (T2DM) has been evolving to introduce other options when intensified treatment is needed after oral antidiabetic drugs (OADs). Currently, many clinical practice guidelines, including the consensus guideline of the American Diabetes Association and the European Association for the Study of Diabetes, recommend glucagon-like peptide-1 receptor agonist (GLP-1RA) as the first-line injectable agent ahead of basal insulin for most patients with T2DM (1–4). Following this change, clinicians are more likely to encounter a question about the subsequent therapy to achieve patients’ glycemic target when GLP-1RA treatment fails. Among the different options, combined therapy of basal insulin and GLP-1RA has the advantages of a lower hypoglycemic risk and more weight reduction with non-inferior potency of glycemic control compared to the basal-bolus insulin regimen (5) or other injectable medications (6). Nonetheless, the addition of basal insulin to GLP-1RA when GLP-1RA treatment fails has not been systematically reviewed.

So far, two different products containing fixed-ratio combinations (FRCs) of basal insulin and GLP-1RA were approved by the US Food and Drug Administration and European Medicines Agency: IGlarLixi, which is a combo of insulin glargine and lixisenatide, and IDegLira, which is a combo of insulin degludec and liraglutide (7–10). Simultaneous delivery of insulin and GLP-1RA with once-daily titration of FRC simplifies the injection and dosing titration compared to the free up-titration approach, which needs a separate injection of basal insulin and GLP-1RA (11). However, it is still unclear whether one of the two methods is superior to the other in its glycemic efficacy or safety, as no randomized controlled trial (RCT) comparing those two approaches has been reported.

Based on these backgrounds, this study aimed to evaluate the overall effect of adding basal insulin on persisting GLP-1RA treatment. Additionally, an indirect comparison between the free up-titration approach of basal insulin and GLP-1RA and switching to FRC was conducted for glycemic efficacy and safety.

## Methods

### Search Strategy and Study Selection

The Preferred Reporting Items for Systematic Reviews and Meta-Analyses checklist was inspected for the systematic review (12). Literature searches were conducted in PubMed, Embase, Web of Science, and the Cochrane Library databases from inception to July 20, 2021. The search strategies for adding basal insulin on GLP-1RA and FRC are described in **Tables S1, S2**, respectively.

Study eligibility was evaluated using the population, intervention, comparison, and outcome protocol. RCTs conducted with patients with T2DM uncontrolled with GLP-1RA were included, and the addition and free up-titration of basal insulin or FRC (intervention group) were compared with continuing GLP-1RA (comparator group). English-language articles, which provided the data on the change in HbA1c from baseline, were eligible. Identified studies were full text screened by two investigators (HJ and CJ) independently whether the trials met the inclusion criteria. Any disagreements were resolved through consensus. This article is based on previously conducted studies and does not contain any new studies with human participants or animals performed by any of the authors.

### Data Extraction and Quality Assessment

The primary outcome was the change in HbA1c from baseline to the end of treatment. The secondary outcomes were the change in fasting plasma glucose (FPG) levels, the proportion of patients reaching HbA1c <7.0% (<53.0 mmol/mol), and the risk of confirmed hypoglycemia. **Table S3** indicates the definitions of confirmed hypoglycemia in the included studies. The outcomes were described using forest plots. The FPG levels presented in only mmol/L were converted to mg/dl according to the following formula: 1 mmol/L = 18.018 mg/dl. Information on the author, publication year, funding sources, and other baseline data, including age, duration of diabetes, the ratio of men to women, body mass index (BMI), HbA1c, FPG levels, and antidiabetic medications at randomization were also gathered. Baseline data were determined at the time of randomization, including trials with a run-in period before randomization. For continuous outcomes, the change in mean value from the randomization to the endpoint of the trial was extracted in each intervention group and comparator together with the variability, such as the SD or SE. Estimated treatment difference between the two groups and 95% CI was used if the mean change or variability of respective groups was unreported. The numbers of events or patients who experienced the events were obtained for binary outcomes. Data extraction was completed by two authors (HJ and CJ) independently according to a predetermined data extraction form.

Individual trials were analyzed for their quality using the Cochrane Collaboration’s tool for assessing the risk of bias in randomized trials (**Figure S1**) (13). Two independent investigators (HJ and YC) evaluated the risk of bias and conducted discussions to resolve different interpretations.

### Statistical Analysis

The pooled estimates of the weighted mean differences (WMDs) and 95% CIs for continuous outcomes, including the changes in HbA1c and FPG, as well as the pooled risk ratios (RRs) and their 95% CIs for dichotomous outcomes, including the proportion of participants achieving target HbA1c values and the risk of hypoglycemia, were calculated. Studies were combined using a random-effects model, and summary results were represented by forest plots. Statistical heterogeneity between studies was evaluated using I^2^ statistics. The potential risk of publication bias was evaluated by constructing funnel plots of the primary outcome (**Figure S2**), with asymmetry assessed by Egger’s test. The validity of the methods for the analysis of indirect comparisons was evaluated, and an indirect estimate of the treatment effect of the free up-titration vs. FRC was determined (14, 15). Stata version 11 software (StataCorp LP, College Station, TX, USA) was used for all statistical analyses.

## Results

### Study Selection

**Figure 1:**
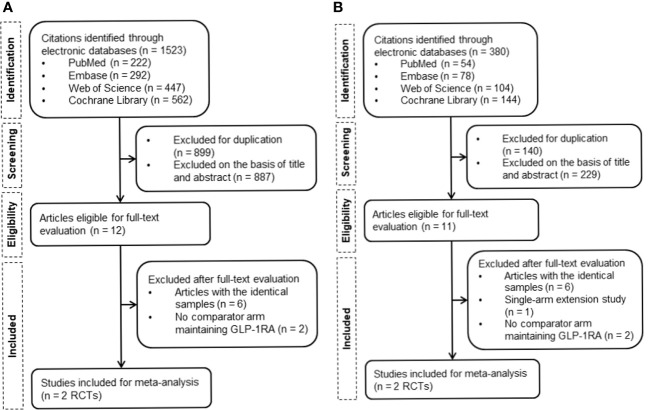
Flowchart of the study selection. **(A)** Flowchart of study retrieval for the addition of basal insulin to GLP-1RA. **(B)** Flowchart of study retrieval for FRC. GLP-1RA, glucagon-like peptide-1 receptor agonist; RCTs, randomized controlled trials; FRC, fixed-ratio combination.

A total of 1,523 and 380 publications were identified through a literature search for the addition of basal insulin on GLP-1RA and FRC, respectively. Four eligible RCTs were finally included in the meta-analysis (**Figure 1**). DeVries et al. (NCT00856986) and Aroda et al. (BEGIN: ADD TO GLP-1 Study; NCT01664247) evaluated the effect of the addition and free up-titration of basal insulin (16, 17), while Linjawi et al. (DUAL III; NCT01676116) and Blonde et al (LixiLan-G; NCT02787551) showed the outcome of switching to FRC (18, 19), all compared to remaining on GLP-1RA.

### Baseline Characteristics

**Table 1** summarizes the study designs and baseline features of the enrolled trials. All studies were randomized, parallel-group, multinational trials sponsored by pharmaceutical industries. The study durations were 26 weeks equally. All trials adopted an open-label design, except for the study of Aroda et al., which was a double-blind trial. Eligibility criteria included adults with T2DM who had not been treated with insulin at least a year before the screening and whose HbA1c was still ≥7.0% at maximal or maximally tolerated GLP-1RA dose. DeVries et al. and Aroda et al. had a run-in period before randomization and withdrew all OADs excluding metformin at the start of the run-in phase (16, 17). In contrast, Linjawi et al. and Blonde et al. maintained previous OADs during the trial (18, 19). Allowable OADs other than metformin were sulfonylurea and/or pioglitazone in the study of Linjawi et al., while they were sodium–glucose cotransporter 2 (SGLT2) and/or pioglitazone in the study of Blonde et al. (18, 19). Liraglutide was the only GLP-1RA used in the study of DeVries et al. and Aroda et al. (16, 17) and was the most frequently used GLP-1RA in other studies (18, 19). The subjects in the study of Linjawi et al. could use GLP-1RA only with a dosing frequency of once or more a day (liraglutide or exenatide twice a day), whereas those in the study of Blonde et al. were permitted to use GLP-1RA with any dosing frequency (18, 19). Approximately 40% of patients in the study of Blonde et al. were on weekly GLP-1RA at randomization (19).

**Table 1:** Outlines of the included studies.

	Free up-titration				FRC			
Author, year (ref)	DeVries, 2012 (16)		Aroda, 2016 (17)		Linjawi, 2017 (18)		Blonde, 2019 (19)	
**Study summary**								
Study design/Duration, weeks	R, P, O/26		R, P, DB/26		R, P, O/26		R, P, O/26	
Intervention/comparator	Insulin detemir + GLP-1RA	GLP-1RA	Insulin degludec + GLP-1RA	GLP-1RA	IDegLira^†^	GLP-1RA	IGlarLixi^‡^	GLP-1RA
	(n = 162)	(n = 161)	(n = 174)	(n = 172)	(n = 292)	(n = 146)	(n = 252)	(n = 253)
Study funder	Novo Nordisk		Novo Nordisk		Novo Nordisk		Sanofi	
**Baseline characteristics**								
Age, years	56.8 (9.4)	57.3 (9.8)	57.0 (10.0)	57.3 (9.4)	58.3 (9.9)	58.4 (8.8)	59.2 (9.6)	60.0 (10.3)
Duration of diabetes, years	8.6 (5.8)	8.5 (6.0)	9.7 (5.8)	9.3 (5.4)	10.4 (5.8)	10.4 (5.8)	11.2 (7.4)	11.0 (6.1)
Male, %	54.3	55.3	56.3	60.5	52.4	48.6	49	56
BMI, kg/m^2^	34.9 (6.3)	33.9 (6.0)	32.0 (5.7)	32.4 (5.4)	32.9 (4.4)	33.0 (4.1)	32.8 (4.4)	33.0 (4.4)
HbA1c, %	7.6 (0.6)	7.6 (0.7)	7.6 (0.6)	7.6 (0.6)	7.8 (0.6)	7.7 (0.6)	7.9 (0.6)	7.9 (0.5)
FPG, mg/dl	166 (34)^§^	159 (38)^§^	156 (38)	164 (40)	162 (38)	169 (42)	163 (38)	170 (35)
OAD at randomization, %								
Metformin only	100.0	100.0	100.0	100.0	74.3	74.0	85.2^¶^	81.3^¶^
Metformin + sulfonylurea	0	0	0	0	20.9	21.9	0	0
Metformin + Pioglitazone	0	0	0	0	2.4	2.7	4.7	8.6
Metformin + SGLT2 inhibitor	0	0	0	0	0	0	10.1	10.1
Metformin + sulfonylurea+ pioglitazone	0	0	0	0	2.4	1.4	0	0
Previous GLP-1RA, %								
Liraglutide	100	100	100	100	79.5	79.5	52.5	56.4
Dulaglutide	0	0	0	0	0	0	21.0	19.8
Exenatide	0	0	0	0	20.5	20.5	7.0	3.5
Exenatide ER	0	0	0	0	0	0	17.5	18.7
Albiglutide	0	0	0	0	0	0	1.9	1.6
Mean duration of GLP-1RA before randomization, weeks	12.0		15.0		66.9		99.1	

Data are expressed in mean (SD) unless otherwise indicated.

FRC, fixed-ratio combination; R, randomized; P, parallel; O, open; DB, double-blind; GLP-1RA, glucagon-like peptide-1 receptor agonists; BMI, body mass index; FPG, fasting plasma glucose; OAD, oral antidiabetic drug; SGLT2, sodium–glucose cotransporter 2; ER, extended release.

^†^FRC comprising of insulin degludec with liraglutide.

^‡^FRC comprising of insulin glargine and lixisenatide.

^§^Presented only in mmol/L and thus converted to mg/dl.

^¶^Not presented in the article but calculated by subtracting the percentages of subjects taking metformin with pioglitazone or metformin with SGLT2 inhibitor from the whole, considering all participants were assigned to one of the three groups; metformin, metformin with pioglitazone, or metformin with SGLT2 inhibitor.

### Dose Titration

Dose adjustment guidelines of basal insulin or FRC used by the included trials are displayed in **Table S4**. Dose titration was based on three preceding values of self-measured plasma glucose at the fasting state in all studies, although target ranges for self-measured plasma glucose were dissimilar. Doses were adjusted with an FPG goal of 72 to 108 mg/dl in the study of DeVries et al., 72 to 90 mg/dl in the study of Aroda et al. and Linjawi et al., and 80 to 100 mg/dl in the study of Blonde et al. (16–19). In the study of DeVries et al., insulin detemir was adjusted from 10 U per day in the beginning to 39.5 U per day (0.41 U/kg), finally, on average (16). The initial dose of insulin degludec was 10 U per day in the study of Aroda et al. and then reached a mean dose of 51 U per day (0.54 U/kg) (17). Linjawi et al. and Blonde et al. started with 16 U per day of IDegLira and 10 U per day of IGlarLixi, respectively (18, 19). The mean doses at the end of the trial in each study were 43 U per day (0.44 U/kg) and 43.5 U per day (0.46 U/kg) (18, 19).

### Glycemic Control

**Figure 2:**
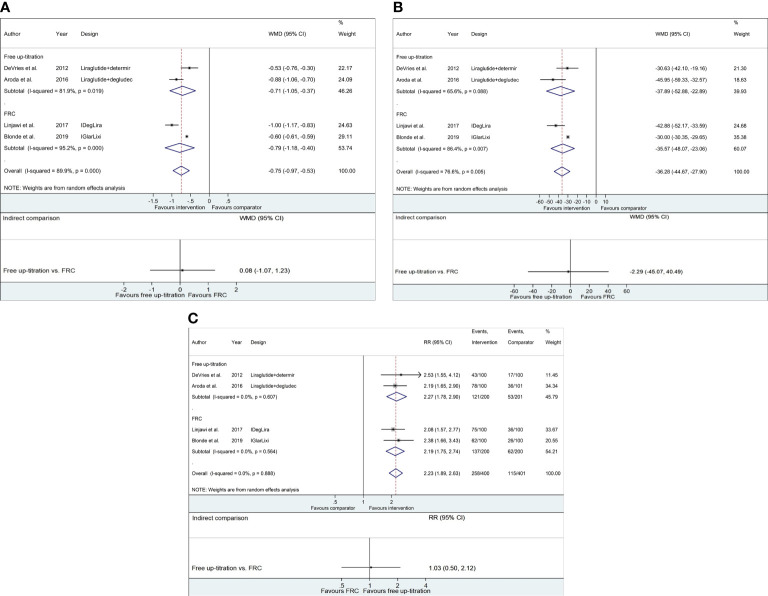
Forest plots of meta-analysis for glycemic control. **(A)** WMDs of HbA1c change (%) from baseline to week 26. **(B)** WMDs of FPG change (mg/dl) from baseline to week 26. **(C)** Relative risks of the fraction of subjects achieving HbA1c < 7.0%. For respective figures, comparisons between the intervention (free up-titration or FRC) and comparator groups (maintaining GLP-1RA) in each trial are described in the top part, whereas indirect comparisons using pooled data are shown in the bottom part. The horizontal lines on both sides of the squares show 95% CI. The diamonds reflect the pooled estimates. WMD, weighted mean difference; RR, relative risk; FPG, fasting plasma glucose.

Compared to maintaining GLP-1RA, both strategies of adding basal insulin (i.e., free-up titration of basal insulin or switching to FRC) effectively decreased HbA1c from baseline to week 26 (free up-titration, WMD −0.71%, 95% CI −1.05 to −0.37; FRC, WMD −0.79%, 95% CI −1.18 to −0.40; **Figure 2A** and **Table S5**). The mean change in HbA1c was not different between the two approaches (WMD 0.08%, 95% CI −1.07 to 1.23). Likewise, the average change in FPG through the trial was −37.89 mg/dl (95% CI −52.88 to −22.89 mg/dl) and −35.57 mg/dl (95% CI −48.07 to −23.06 mg/dl) for free up-titration and FRC, respectively, causing insignificant difference (WMD −2.29 mg/dl, 95% CI −45.07 to 40.49 mg/dl; **Figure 2B**). The percentage of achieving HbA1c < 7% was also higher for adding basal insulin than for unchanged GLP-1RA (RR 2.23, 95% CI 1.89−2.63) but similar between the free up-titration and FRC (RR 1.03, 95% CI 0.50−2.14; **Figure 2C**).

### Hypoglycemia

**Figure 3:**
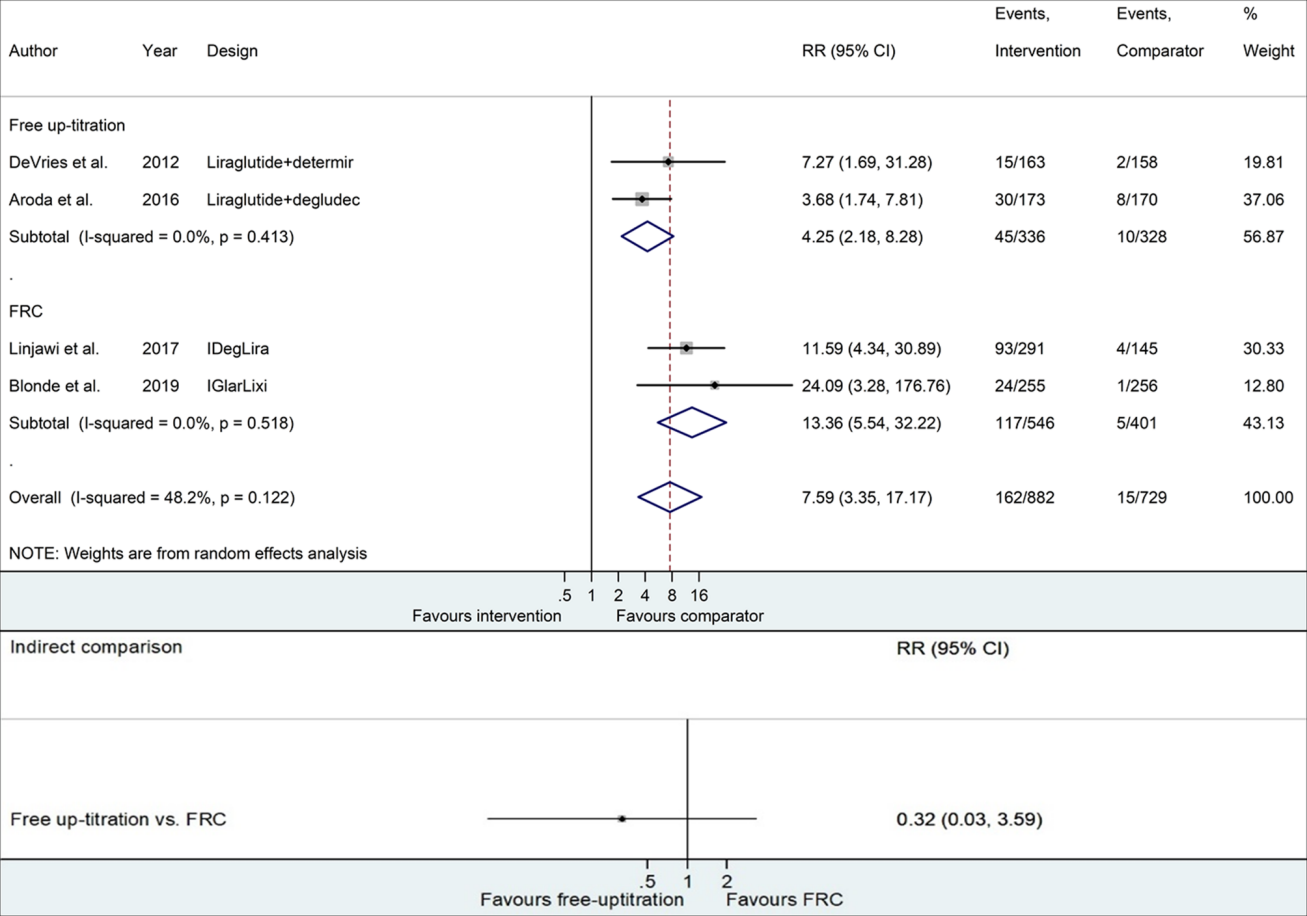
Forest plots of meta-analysis for the risk of confirmed hypoglycemia. Comparisons between the intervention (free up-titration or FRC) and comparator groups (maintaining GLP-1RA) in each trial are described in the top part, whereas indirect comparison using pooled data is shown in the bottom part. The horizontal lines on both sides of the squares show 95% CI. The diamonds reflect the pooled estimates. RR, relative risk; FRC, fixed-ratio combination; GLP-1RA, glucagon-like peptide-1 receptor agonist.

**Figure 3** and **Table S5** present the risk of confirmed hypoglycemia with the addition of basal insulin compared to continuing GLP-1RA. The free up-titration of insulin presented a 4.25 times higher risk of hypoglycemia than the comparator (95% CI 2.18−8.28), while FRC displayed 13.36 times higher risk (95% CI 5.54−32.22). However, no statistical significance was confirmed for the difference between the two methods (RR 0.32, 95% CI 0.03−3.59).

The difference in the weight change could not be calculated because Aroda et al. (17) did not report the variability for each group of intervention and comparator nor estimate the treatment difference between the two groups. Also, as most trials had no event of severe hypoglycemia, making it impossible to compute RR, the comparison for severe hypoglycemia was not displayed.

## Discussion

A meta-analysis of four RCTs evaluating the efficacy and safety of adding basal insulin in T2DM subjects insufficiently managed using GLP-1RA showed that the free up-titration of basal insulin and GLP-1RA and switching to FRC effectively enhanced glycemic control compared to unchanged GLP-1RA but accompanied elevated hypoglycemic risk. Specifically, compared with persisting GLP-1RA, the addition of basal insulin by either free up-titration or FRC caused an additional decrease of 0.75% and 36.3 mg/dl in HbA1c and FPG, respectively, and a 7.59 times higher risk of hypoglycemia in 26 weeks. An indirect comparison of the two approaches, however, showed no significant difference in their glycemic efficacy and hypoglycemic risk.

Since 2019, the American Diabetes Association “Standards of Medical Care in Diabetes” advocates GLP-1RA before basal insulin when injectable therapy is needed (20). Additionally, it recommends that clinicians intensify treatment by adding basal insulin subsequently in patients who failed to reach target HbA1c levels despite the GLP-1RA treatment (2). A meta-analysis of 15 RCTs contrasting the combination of GLP-1RA and basal insulin with other antidiabetic agents showed 0.44% and 3.22 kg greater decrease in HbA1c and body weight, respectively, by combining GLP-1RA and basal insulin without increasing hypoglycemic risk (21). Interestingly, compared with the basal-bolus insulin regimen, the combination of GLP-1RA and basal insulin displayed a similar benefit in lowering HbA1c with a 33% lower risk of hypoglycemia and 5.66 kg less body weight gain (21). Similarly, Maiorino et al. analyzed the effects of the combination treatment of basal insulin and GLP-1RA relative to other injectable antidiabetics through a meta-analysis of 26 RCTs involving 11,425 patients (6). GLP-1RA plus basal insulin decreased HbA1c by 0.47% more than other injectable strategies combined, yielding 1.65-fold higher percentages of patients achieving HbA1c < 7.0% (6). Although the combo therapy was not superior in reducing HbA1c compared to basal-bolus insulin regimens, it indicated a 34% lower risk of hypoglycemia and 4.7 kg more weight reduction (6). These findings propose the use of basal insulin combined with GLP-1RA as the best therapeutic choice for patients who failed to reach target HbA1c levels despite the GLP-1RA treatment. However, most trials included in previous meta-analyses added GLP-1RA to the background insulin therapy, which only indirectly reflects the advantage of combination treatment in the setting of GLP-1RA failure. Our study is novel in that it consists of only RCTs with insulin addition in patients inadequately controlled with GLP-1RA, which conforms to the present clinical guidelines where GLP-1RA is generally recommended as the first injectable (1–4).

DeVries et al. (16) and Aroda et al. (17) showed respectively 0.53% and 0.88% more reduction in HbA1c with the free up-titration of basal insulin and GLP-1RA relative to unchanged GLP-1RA, while Linjawi et al. (18) and Blonde et al. (19) exhibited respectively 1% and 0.6% more reduction in HbA1c by comparing switching to FRC with continuing GLP-1RA. However, the preferable method of adding basal insulin to GLP-1RA among free up-titration and FRC has not yet been determined. Contrary to the free combinations of basal insulin and GLP-1RA, FRC eschews the need for a separate GLP-1RA injection apart from the daily administration of basal insulin (11). Alternatively, FRC lacks the titrating flexibility of each medication according to problematic glucose trends, such as exceptionally increasing fasting or postprandial glucose (22). Furthermore, the maximal insulin doses are limited for FRC, although the greatest dose of 50 U for IDegLira and 60 U for IGlarLixi seems fairly acceptable for most patients (7, 9).

A recent meta-analysis reported comparing each strategy of combo therapy with the up-titration of basal insulin, showing no significant difference between the two approaches in HbA1c change, target HbA1c achievement rate, hypoglycemic risk, and body weight change (23). Similarly, in a recent Italian multicenter retrospective study, HbA1c reduction was also similar between the free up-titration and FRC (24). However, this real-world study presented fewer final insulin doses and greater weight loss in the free up-titration group (24). The risk of hypoglycemia was not evaluated in this study (24). The present analysis found that the efficacy of glycemic control was comparable between the free up-titration and FRC, which corresponds to the previous studies that contrasted the two methods (23, 24). Collectively, FRC may be a beneficial choice for improving adherence by reducing the number of injections with similar glycemic achievement compared to the free up-titration. Nevertheless, the dissimilar criteria for comparator or background medications in this study and the antecedent reports should be recognized. The comparator of the former meta-analysis was basal insulin intensification (23), and the latter multicenter retrospective study was also composed of T2DM patients using basal insulin beforehand (24).

Meanwhile, GLP-1RAs combined with each of the two FRC products show different temporal actions despite the same dosing frequency: lixisenatide of IGlarLixi is a short-acting agent, while liraglutide of IDegLira is a long-acting agent. A systematic review was performed recently to compare the effects of short-acting and long-acting GLP-1RAs, both in combination with basal insulin (25). It reported a superior reduction of FPG, HbA1c, and body weight by long-acting agents with a lower incidence of symptomatic hypoglycemia and gastrointestinal adverse reactions (25). On the contrary, delayed gastric emptying by GLP-1RA was more preserved with short-acting agents (26, 27). The differential actions of lixisenatide and liraglutide might affect the outcomes of FRCs containing each GLP-1RA, which need to be clarified in future research.

Several limitations should be considered for the interpretation of this study. First, only four trials were selected for the meta-analysis. The paucity of suitable trials reflects the requirement for further evidence on the preferred regimen following GLP-1RA failure. Second, outcomes for comparison were restricted to HbA1c, FPG, and hypoglycemic risk, which were presented in all included trials. Additional assessment of body weight change or self-measured plasma glucose, along with the analysis of each change in fasting and postprandial glucose, would be clinically beneficial. Third, there was heterogeneity between the included trials, such as study design, the definition of confirmed hypoglycemia, dose titration strategy, and antidiabetic medication used at baseline. Specifically, Aroda et al. was the only double-blind trial (17). The threshold for defining hypoglycemia was a plasma glucose level of below 56 mg/dl in the study of DeVries et al. and Aroda et al., 56 mg/dl or lower in the study of Linjawi et al., and below 54 mg/dl in the study of Blonde et al. (16–19). Target FPG for dose adjustment was also marginally different between trials (16–19). Only metformin and liraglutide were permitted at randomization in the study of DeVries et al. and Aroda et al., while Linjawi et al. and Blonde et al. allowed other OADs and GLP-1RAs with various dosing frequencies as well (16–19). However, metformin was maintained in all studies, and the most frequently prescribed GLP-1RA was liraglutide. The duration of GLP-1RA treatment before adding basal insulin was also inconsistent in selected trials (**Table 1**). It is assumed to influence successful glycemic control, as indicated by a real-world study of 66,583 patients with T2DM, which demonstrated a higher proportion of patients accomplishing HbA1c < 7% with insulin addition within 6 months of beginning GLP-1RA compared to later addition (28). Despite the constraints, this study is valuable for being the first meta-analysis that directly evaluated the benefit and risk of basal insulin addition and compared the free up-titration and FRC in the context of ongoing GLP-1RA.

## Conclusion

The addition of basal insulin by either free up-titration or FRC efficiently improves glycemic control but increases hypoglycemic risk in patients with T2DM whose glycemic targets were unmet with GLP-1RA. The efficacy and safety appear to be equivalent between the two methods, even though the interpretation is limited by the small numbers and heterogeneity of selected trials. Further randomized studies are warranted to contrast the free up-titration and FRC for various outcomes in patients inadequately controlled with GLP-1RA.

## Author Contributions

HJ and YC: data analysis, interpretation, and original draft preparation. SM, HK, Y-JK, J-YP, and WL: data interpretation, review, and editing. CJ: supervision, conceptualization, review, and editing. All authors listed have made a substantial, direct, and intellectual contribution to the work and approved it for publication.

## Conflict of Interest

The authors declare that the research was conducted in the absence of any commercial or financial relationships that could be construed as a potential conflict of interest.

## Publisher’s Note

All claims expressed in this article are solely those of the authors and do not necessarily represent those of their affiliated organizations, or those of the publisher, the editors and the reviewers. Any product that may be evaluated in this article, or claim that may be made by its manufacturer, is not guaranteed or endorsed by the publisher.

## Abbreviations

T2DM, type 2 diabetes mellitus; OAD, oral antidiabetic drug; GLP-1RA, glucagon-like peptide-1 receptor agonist; FRC, fixed-ratio combination; RCT, randomized controlled trial; FPG, fasting plasma glucose; BMI, body mass index; WMD, weighted mean difference; RR, risk ratios.
