# Supplementary material for: Free Versus Fixed-Ratio Combination of Basal Insulin and GLP-1 Receptor Agonists in Type 2 Diabetes Uncontrolled With GLP-1 Receptor Agonists: A Systematic Review and Indirect Treatment Comparison
Source: Front Endocrinol (Lausanne). 2022 May 20;13:870722. doi: 10.3389/fendo.2022.870722 (PMC9165059; doi:10.3389/fendo.2022.870722)
Supplement: Supplementary file 1 [file DataSheet_1.docx]

Supplementary Material

**Contents**

**Table S1.** Search strategy for the addition of basal insulin on GLP-1RA

**Table S2.** Search strategy for FRC

**Table S3.** Definitions of confirmed hypoglycemia in the selected trials

**Table S4.** Titration guidelines of insulin or FRC used in the selected trials

**Table S5.** The overall and respective outcomes of the free up-titration of basal insulin and FRC compared to maintaining GLP-1RA

**Figure S1.** Cochrane risk of bias in the selected trials.

**Figure S2.** Funnel plots for absolute HbA1c changes in the included studies.

**PRISMA checklist**

**PICO protocol**

**Table S1.** Search strategy for the addition of basal insulin on GLP-1RA

| **Data source** | **Search terms** |  |  |
| --- | --- | --- | --- |
| **PubMed** | #1 "Diabetes Mellitus"[Mesh:NoExp] OR "diabetes mellitus, type 2"[MeSH]  #2 NIDDM[TW]  #3 ("type 2"[TW] OR "Type2"[TW] OR Type II[TW] OR TypeII[TW] OR Maturity-Onset[TW] OR "Maturity Onset"[TW] OR Adult-Onset[TW] OR Ketosis-Resistant[TW] OR "Ketosis Resistant"[TW] OR "Non Insulin"[TW] OR Non-Insulin[TW] OR Noninsulin[TW]) AND diabet*[TW]  #4 #1 OR #2 OR #3  #5 "Glucagon-Like Peptide 1"[Mesh] OR "Glucagon-Like Peptide-1 Receptor"[Mesh] OR "Glucagon Like Peptide 1"[TW] OR "Glucagon-Like Peptide-1"[TW] OR "GLP-1"[TW] OR "GLP 1"[TW]  #6 Liraglutide[Mesh] OR "Liraglutide"[TW] OR Victoza[TW] OR Saxenda[TW] OR "NN2211"[TW] OR "NN 2211"[TW] OR "NN-2211"[TW]  #7 "exenatide" [Supplementary Concept] OR exenatide[TW] OR Byetta[TW] OR "exendin-4"[TW] OR "exendin 4"[TW] OR bydureon[TW]  #8 "semaglutide" [Supplementary Concept] OR semaglutide[TW]  #9 "dulaglutide" [Supplementary Concept] OR dulaglutide[TW] OR Trulicity[TW]  #10 "rGLP-1 protein" [Supplementary Concept] OR albiglutide[TW] OR Eperzan[TW] OR Tanzeum[TW]  #11 #5 OR #6 OR #7 OR #8 OR #9 OR #10  #12 Basal[TW] AND ("Insulin"[Mesh] OR "Insulin"[TW])  #13 "Insulin Glargine"[Mesh] OR Glargine[TW] OR Lantus[TW] OR Basaglar[TW] OR toujeo[TW]  #14 "Insulin Detemir"[Mesh] OR Detemir[TW] OR Levemir[TW]  #15 "insulin degludec" [Supplementary Concept] OR degludec[TW] OR tresiba[TW]  #16 #12 OR #13 OR #14 OR #15  #17 #4 AND #11 AND #16  #18 #17 AND (Randomized Controlled Trial[ptyp] OR Randomized[TI] OR Randomised[TI] OR Randomly[TI] OR RCT[TI] OR Placebo[TI] OR ((Double*[TI] OR single*[TI] OR treb*[TI] OR tripl*[TI]) AND (Blind*[TI] OR mask*[TI])) OR random*[TI])  #19 #18 NOT (animals[Mesh:noexp] NOT (animals[Mesh:noexp] AND humans[Mesh])) AND English[Lang] | **Embase** | #1 'non insulin dependent diabetes mellitus'/exp  #2 ('non insulin dependent diabetes mellitus' OR niddm):ab,ti,kw  #3 (diabet* NEAR/6 ('type 2' OR 'type2' OR 'type ii' OR 'typeii' OR 'maturity-onset' OR 'maturity onset' OR 'adult-onset' OR 'ketosis-resistant' OR 'ketosis resistant' OR 'non insulin' OR 'non-insulin' OR noninsulin)):ab,ti,kw  #4 #1 OR #2 OR #3  #5 ''glucagon like peptide 1'/exp OR 'glucagon like peptide 1 receptor agonist'/exp OR ('Glucagon Like Peptide 1' OR 'Glucagon-Like Peptide-1' OR 'GLP-1' OR 'GLP 1'):ab,ti,kw  #6 ''liraglutide'/exp OR (liraglutide OR Victoza OR Saxenda OR 'NN2211' OR 'NN 2211' OR 'NN-2211'):ab,ti,kw  #7 ''exendin 4'/exp OR (exenatide OR Byetta OR 'exendin-4' OR 'exendin 4' OR bydureon):ab,ti,kw  #8 ''semaglutide'/exp OR semaglutide:ab,ti,kw  #9 ''dulaglutide'/exp OR (dulaglutide OR Trulicity):ab,ti,kw  #10 ''albiglutide'/exp OR ('rGLP-1 protein' OR albiglutide OR Eperzan OR Tanzeum):ab,ti,kw  #11 #5 OR #6 OR #7 OR #8 OR #9 OR #10  #12 Basal AND ('insulin'/exp OR 'Insulin')  #13 ''insulin glargine'/exp OR (Glargine OR Lantus OR Basaglar OR toujeo):ab,ti,kw  #14 ''insulin detemir'/exp OR (Detemir OR Levemir):ab,ti,kw  #15 ''insulin degludec'/exp OR (degludec OR tresiba):ab,ti,kw  #16 #12 OR #13 OR #14 OR #15  #17 #4 AND #11 AND #16  #18 #17 AND ([randomized controlled trial]/lim OR 'randomized controlled trial'/exp OR 'double blind procedure'/exp OR 'single blind procedure'/exp OR 'triple blind procedure'/exp OR randomized:ti OR randomised:ti OR random*:ti OR placebo:ti)  #19 #18 NOT ('animal'/de NOT ('animal'/de AND 'human'/exp)) AND [english]/lim  #20 #19 AND ([article]/lim OR [article in press]/lim) |
| **Web of Science** | #1 TS=(("type 2" OR "Type2" OR "Type II" OR "TypeII" OR "Maturity Onset" OR "Adult Onset" OR "Ketosis Resistant" OR "Non Insulin" OR "Noninsulin") NEAR/6 diabet*)  #2 TS=(NIDDM)  #3 #1 OR #2  #4 TS=("Glucagon Like Peptide 1" OR "Glucagon-Like Peptide-1" OR "GLP-1" OR "GLP 1")  #5 TS=("Liraglutide" OR Victoza OR Saxenda OR "NN2211" OR "NN 2211" OR "NN-2211")  #6 TS=(exenatide OR Byetta OR "exendin-4" OR "exendin 4" OR bydureon)  #7 TS=(semaglutide)  #8 TS=(dulaglutide OR Trulicity)  #9 TS=(albiglutide OR Eperzan OR Tanzeum)  #10 #4 OR #5 OR #6 OR #7 OR #8 OR #9  #11 TS=(Basal AND "Insulin")  #12 TS=(Glargine OR Lantus OR Basaglar OR toujeo)  #13 TS=(Detemir OR Levemir)  #14 TS=(degludec OR tresiba)  #15 #11 OR #12 OR #13 OR #14  #16 #3 AND #10 AND #15  #17 #16 AND TS=(random* OR placebo* OR RCT)  #18 #17 Refined by: DOCUMENT TYPES: ( ARTICLE OR EARLY ACCESS), Language; English | **Cochrane** | #1 [mh ^"Diabetes Mellitus"] OR [mh "diabetes mellitus, type 2"]  #2 NIDDM  #3 ("type 2" OR "Type2" OR Type II OR TypeII OR Maturity-Onset OR "Maturity Onset" OR Adult-Onset OR Ketosis-Resistant OR "Ketosis Resistant" OR "Non Insulin" OR Non-Insulin OR Noninsulin) NEAR/6 diabet*  #4 #1 OR #2 OR #3  #5 [mh "Glucagon-Like Peptide 1"] OR [mh "Glucagon-Like Peptide-1 Receptor"] OR "Glucagon Like Peptide 1" OR "Glucagon-Like Peptide-1" OR "GLP-1" OR "GLP 1"  #6 [mh "Liraglutide"] OR "Liraglutide" OR Victoza OR Saxenda OR "NN2211" OR "NN 2211" OR "NN-2211"  #7 exenatide OR Byetta OR "exendin-4" OR "exendin 4" OR bydureon  #8 semaglutide  #9 dulaglutide OR Trulicity  #10 albiglutide OR Eperzan OR Tanzeum  #11 #5 OR #6 OR #7 OR #8 OR #9 OR #10  #12 Basal AND ([mh "Insulin"] OR "Insulin")  #13 [mh "Insulin Glargine"] OR Glargine OR Lantus OR Basaglar OR toujeo  #14 [mh "Insulin Detemir"] OR Detemir OR Levemir  #15 degludec OR tresiba  #16 #12 OR #13 OR #14 OR #15  #17 #4 AND #11 AND #16  #18 #17 in Trials (exclude ongoing trial, Conference paper) |

Abbreviations: GLP-1RA, glucagon-like peptide-1 receptor agonists.

**Table S2.** Search strategy for FRC

| **Data source** | **Search terms** |  |  |
| --- | --- | --- | --- |
| **PubMed** | #1 "Diabetes Mellitus"[Mesh:NoExp] OR "diabetes mellitus, type 2"[MeSH]  #2 NIDDM[TW]  #3 ("type 2"[TW] OR "Type2"[TW] OR Type II[TW] OR TypeII[TW] OR Maturity-Onset[TW] OR "Maturity Onset"[TW] OR Adult-Onset[TW] OR Ketosis-Resistant[TW] OR "Ketosis Resistant"[TW] OR "Non Insulin"[TW] OR Non-Insulin[TW] OR Noninsulin[TW]) AND diabet*[TW]  #4 #1 OR #2 OR #3  #5 "Glucagon-Like Peptide 1"[Mesh] OR "Glucagon-Like Peptide-1 Receptor"[Mesh] OR "Glucagon Like Peptide 1"[TW] OR "Glucagon-Like Peptide-1"[TW] OR "GLP-1"[TW] OR "GLP 1"[TW]  #6 Liraglutide[Mesh] OR "Liraglutide"[TW] OR Victoza[TW] OR Saxenda[TW] OR "NN2211"[TW] OR "NN 2211"[TW] OR "NN-2211"[TW]  #7 "exenatide" [Supplementary Concept] OR exenatide[TW] OR Byetta[TW] OR "exendin-4"[TW] OR "exendin 4"[TW] OR bydureon[TW]  #8 "semaglutide" [Supplementary Concept] OR semaglutide[TW]  #9 "dulaglutide" [Supplementary Concept] OR dulaglutide[TW] OR Trulicity[TW]  #10 "rGLP-1 protein" [Supplementary Concept] OR albiglutide[TW] OR Eperzan[TW] OR Tanzeum[TW]  #11 #5 OR #6 OR #7 OR #8 OR #9 OR #10  #12 fixed-ratio[TW] AND combination*[TW]  #13 IDegLira[TW]  #14 ("insulin degludec" [Supplementary Concept] OR degludec[TW] OR tresiba[TW]) AND (Liraglutide[Mesh] OR "Liraglutide"[TW] OR Victoza[TW] OR Saxenda[TW] OR "NN2211"[TW] OR "NN 2211"[TW] OR "NN-2211"[TW])  #15 IGlarLixi[TW]  #16 ("Insulin Glargine"[Mesh] OR Glargine[TW] OR Lantus[TW] OR Basaglar[TW] OR toujeo[TW]) AND ("ZP10A peptide" [Supplementary Concept] OR lixisenatide[TW] OR Lyxumia[TW] OR Lixisenatide[TW] OR "AVE0010"[TW] OR Adlyxin[TW])  #17 #12 OR #13 OR #14 OR #15 OR #16  #18 #4 AND #11 AND #17  #19 #18 AND (Randomized Controlled Trial[ptyp] OR Randomized[TI] OR Randomised[TI] OR Randomly[TI] OR RCT[TI] OR Placebo[TI] OR ((Double*[TI] OR single*[TI] OR treb*[TI] OR tripl*[TI]) AND (Blind*[TI] OR mask*[TI])) OR random*[TI])  #20 #19 NOT (animals[Mesh:noexp] NOT (animals[Mesh:noexp] AND humans[Mesh])) AND English[Lang] | **Embase** | #1 'non insulin dependent diabetes mellitus'/exp  #2 ('non insulin dependent diabetes mellitus' OR niddm):ab,ti,kw  #3 (diabet* NEAR/6 ('type 2' OR 'type2' OR 'type ii' OR 'typeii' OR 'maturity-onset' OR 'maturity onset' OR 'adult-onset' OR 'ketosis-resistant' OR 'ketosis resistant' OR 'non insulin' OR 'non-insulin' OR noninsulin)):ab,ti,kw  #4 #1 OR #2 OR #3  #5 ''glucagon like peptide 1'/exp OR 'glucagon like peptide 1 receptor agonist'/exp OR ('Glucagon Like Peptide 1' OR 'Glucagon-Like Peptide-1' OR 'GLP-1' OR 'GLP 1'):ab,ti,kw  #6 ''liraglutide'/exp OR (liraglutide OR Victoza OR Saxenda OR 'NN2211' OR 'NN 2211' OR 'NN-2211'):ab,ti,kw  #7 ''exendin 4'/exp OR (exenatide OR Byetta OR 'exendin-4' OR 'exendin 4' OR bydureon):ab,ti,kw  #8 ''semaglutide'/exp OR semaglutide:ab,ti,kw  #9 ''dulaglutide'/exp OR (dulaglutide OR Trulicity):ab,ti,kw  #10 ''albiglutide'/exp OR ('rGLP-1 protein' OR albiglutide OR Eperzan OR Tanzeum):ab,ti,kw  #11 #5 OR #6 OR #7 OR #8 OR #9 OR #10  #12 (fixed-ratio NEAR/6 combination*):ab,ti,kw  #13 ''insulin degludec plus liraglutide'/exp OR IDegLira:ab,ti,kw  #14 ('insulin degludec'/exp OR (degludec OR tresiba):ab,ti,kw) AND ('liraglutide'/exp OR (liraglutide OR Victoza OR Saxenda OR 'NN2211' OR 'NN 2211' OR 'NN-2211'):ab,ti,kw)  #15 ''insulin glargine plus lixisenatide'/exp OR IGlarLixi:ab,ti,kw  #16 ('insulin glargine'/exp OR (Glargine OR Lantus OR Basaglar OR toujeo):ab,ti,kw) AND ('lixisenatide'/exp OR (lixisenatide OR Lyxumia OR Lixisenatide OR "AVE0010" OR Adlyxin):ab,ti,kw)  #17 #12 OR #13 OR #14 OR #15 OR #16  #18 #4 AND #11 AND #17  #19 #18 AND ([randomized controlled trial]/lim OR 'randomized controlled trial'/exp OR 'double blind procedure'/exp OR 'single blind procedure'/exp OR 'triple blind procedure'/exp OR randomized:ti OR randomised:ti OR random*:ti OR placebo:ti)  #20 #19 NOT ('animal'/de NOT ('animal'/de AND 'human'/exp)) AND [english]/lim  #21 #20 AND ([article]/lim OR [article in press]/lim) |
| **Web of Science** | #1 TS=(("type 2" OR "Type2" OR "Type II" OR "TypeII" OR "Maturity Onset" OR "Adult Onset" OR "Ketosis Resistant" OR "Non Insulin" OR "Noninsulin") NEAR/6 diabet*)  #2 TS=(NIDDM)  #3 #1 OR #2  #4 TS=("Glucagon Like Peptide 1" OR "Glucagon-Like Peptide-1" OR "GLP-1" OR "GLP 1")  #5 TS=("Liraglutide" OR Victoza OR Saxenda OR "NN2211" OR "NN 2211" OR "NN-2211")  #6 TS=(exenatide OR Byetta OR "exendin-4" OR "exendin 4" OR bydureon)  #7 TS=(semaglutide)  #8 TS=(dulaglutide OR Trulicity)  #9 TS=(albiglutide OR Eperzan OR Tanzeum)  #10 #4 OR #5 OR #6 OR #7 OR #8 OR #9  #11 TS=(fixed-ratio NEAR/6 combination*)  #12 TS=(IDegLira)  #13 TS=((degludec OR tresiba) AND ("Liraglutide" OR Victoza OR Saxenda OR "NN2211" OR "NN 2211" OR "NN-2211"))  #14 TS=(IGlarLixi)  #15 TS=((Glargine OR Lantus OR Basaglar OR toujeo) AND (lixisenatide OR Lyxumia OR Lixisenatide OR "AVE0010" OR Adlyxin))  #16 #11 OR #12 OR #13 OR #14 OR #15  #17 #3 AND #10 AND #16  #18 #17 AND TS=(random* OR placebo* OR RCT)  #19 #18 Refined by: DOCUMENT TYPES: ( ARTICLE OR EARLY ACCESS), Language; English | **Cochrane** | #1 [mh ^"Diabetes Mellitus"] OR [mh "diabetes mellitus, type 2"]  #2 NIDDM  #3 ("type 2" OR "Type2" OR Type II OR TypeII OR Maturity-Onset OR "Maturity Onset" OR Adult-Onset OR Ketosis-Resistant OR "Ketosis Resistant" OR "Non Insulin" OR Non-Insulin OR Noninsulin) NEAR/6 diabet*  #4 #1 OR #2 OR #3  #5 [mh "Glucagon-Like Peptide 1"] OR [mh "Glucagon-Like Peptide-1 Receptor"] OR "Glucagon Like Peptide 1" OR "Glucagon-Like Peptide-1" OR "GLP-1" OR "GLP 1"  #6 [mh "Liraglutide"] OR "Liraglutide" OR Victoza OR Saxenda OR "NN2211" OR "NN 2211" OR "NN-2211"  #7 exenatide OR Byetta OR "exendin-4" OR "exendin 4" OR bydureon  #8 semaglutide  #9 dulaglutide OR Trulicity  #10 albiglutide OR Eperzan OR Tanzeum  #11 #5 OR #6 OR #7 OR #8 OR #9 OR #10  #12 fixed-ratio NEAR/6 combination*  #13 IDegLira  #14 (degludec OR tresiba) AND ([mh "Liraglutide"] OR "Liraglutide" OR Victoza OR Saxenda OR "NN2211" OR "NN 2211" OR "NN-2211")  #15 IGlarLixi  #16 ([mh "Insulin Glargine"] OR Glargine OR Lantus OR Basaglar OR toujeo) AND (lixisenatide OR Lyxumia OR Lixisenatide OR "AVE0010" OR Adlyxin)  #17 #12 OR #13 OR #14 OR #15 OR #16  #18 #4 AND #11 AND #17  #19 #18 in Trials (exclude ongoing trial, Conference paper) |

Abbreviations: FRC, fixed-ratio combination.

**Table S3.** Definitions of confirmed hypoglycemia in the selected trials

| **Study group** | **Author (year)** | **Definitions of hypoglycemia** |
| --- | --- | --- |
| **Free up-titration** | **DeVries (2012)** | Plasma glucose <3.1 mmol/L (<56 mg/dL) |
|  | **Aroda (2016)** | Asymptomatic and symptomatic episodes confirmed by a measured plasma glucose <3.1 mmol/L (<56 mg/dL) or severe episodes requiring assistance |
| **FRC** | **Linjawi (2017)** | Plasma glucose ≤3.1 mmol/L (≤56 mg/dL) or severe hypoglycemia which required third-party assistance |
|  | **Blonde (2019)** | Plasma glucose <3.0 mmol/L (<54 mg/dL) symptomatic hypoglycemia |

Abbreviations: FRC, fixed-ratio combination.

**Table S4.** Titration guidelines of insulin or FRC used in the selected trials

| **Study group** | **Author (year)** | **Start dose** | **Adjustment methods** | **Target** | **Dose reduction** |
| --- | --- | --- | --- | --- | --- |
| **Free up-titration** | **DeVries (2012)** | Insulin detemir 10 U | Based on SMPG at fasting state on 3 days in the week prior to dose titration, adjusted once a week | 72–108 mg/dL  (4.0–6.0 mmol/L) | Major hypoglycemic episodes and/or FPG <72 mg/dL  (<4.0 mmol/L) without obvious explanation |
|  | **Aroda (2016)** | Insulin degludec 10 U | Based on the average pre-breakfast SMPG on 3 consecutive days | 72–90 mg/dL  (4.0–5.0 mmol/L) | Fasting plasma glucose  <72 mg/dL (<4.0 mmol/L) |
| **FRC** | **Linjawi (2017)** | IDegLira 16 U  (IDeg 16 U and  Lira 0.6 mg) | Based on 3 preceding pre-breakfast SMPG, adjusted twice weekly, maximum of 50 dose steps | 72–90 mg/dL  (4.0–5.0 mmol/L) | FPG <72 mg/dL (<4.0 mmol/L) |
|  | **Blonde (2019)** | IGlarLixi 10 U with 10-40-U pen (IGlar 10 U and Lixi 5 μg) | Based on the lowest fasting SMPG from the last 3 values, adjusted twice weekly during the first 8 weeks and once a week afterward, switched to 30-60-U pen (IGlar 3 U:Lixi 1 μg) if insulin dose exceeds 40 U | 80–100 mg/dL  (4.4–5.6 mmol/L) | FPG <80 mg/dL (<4.4 mmol/L) |

Abbreviations: FRC, fixed-ratio combination; SMPG, self-measured plasma glucose measurements; FPG, fasting plasma glucose; IDeg, insulin degludec; Lira, liraglutide; IGlar, insulin glargine; Lixi, lixisenatide.

**Table S5.** The overall and respective outcomes of the free up-titration of basal insulin and FRC compared to maintaining GLP-1RA

|  | **Intervention/comparator (n)** | **Estimate (95% CI)** | **I^2^** |
| --- | --- | --- | --- |
| **HbA1c change, %** |  | WMD | 89.9 |
| Overall | 880/732 | −0.75 (−0.97, −0.53) |  |
| Free up-titration | 336/333 | −0.71 (−1.05, −0.37) |  |
| FRC | 544/399 | −0.79 (−1.18, −0.40) |  |
| **FPG change, mg/dL** |  | WMD | 76.6 |
| Overall | 880/732 | −36.28 (−44.67, −27.90) |  |
| Free up-titration | 336/333 | −37.89 (−52.88, −22.89) |  |
| FRC | 544/399 | −35.57 (−48.07, −23.06) |  |
| **HbA1c <7%** |  | RR | 0.0 |
| Overall | 880/732 | 2.23 (1.89, 2.63) |  |
| Free up-titration | 336/333 | 2.27 (1.78, 2.90) |  |
| FRC | 544/399 | 2.19 (1.75, 2.74) |  |
| **Confirmed hypoglycemia** |  | RR | 48.2 |
| Overall | 880/732 | 7.59 (3.35, 17.17) |  |
| Free up-titration | 336/333 | 4.25 (2.18, 8.28) |  |
| FRC | 544/399 | 13.36 (5.54, 32.22) |  |

Abbreviations: FRC, fixed-ratio combination; GLP-1RA, glucagon-like peptide-1 receptor agonists; CI, confidence interval; WMD, weighted mean difference; FPG, fasting plasma glucose; RR, relative risk.

**Figure S1.** Cochrane risk of bias in the selected trials


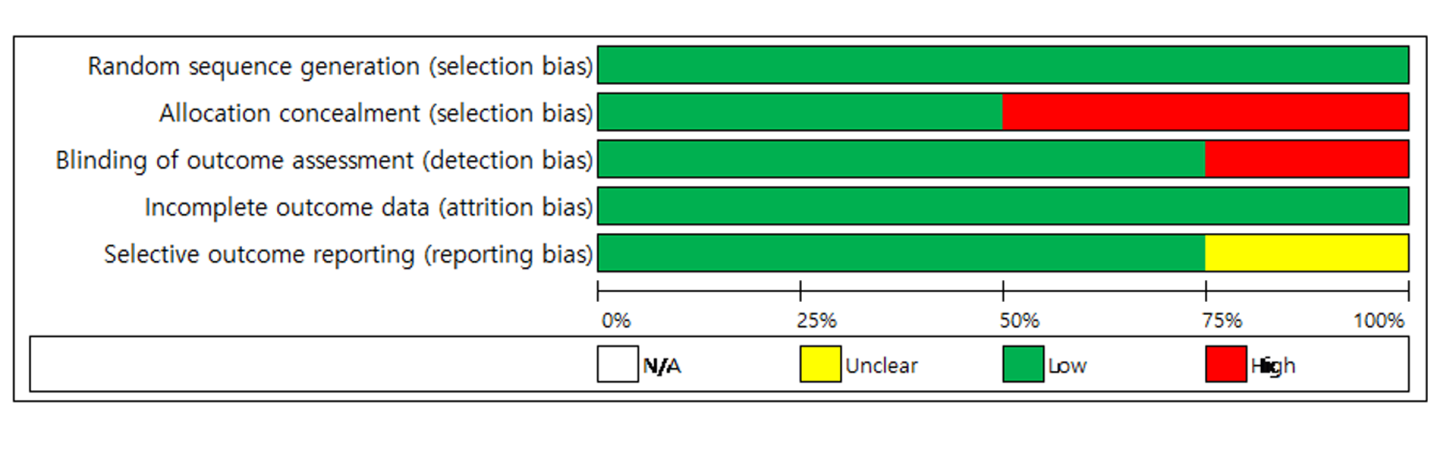


**Figure S2.** Funnel plots for absolute HbA1c changes in the included studies.

**
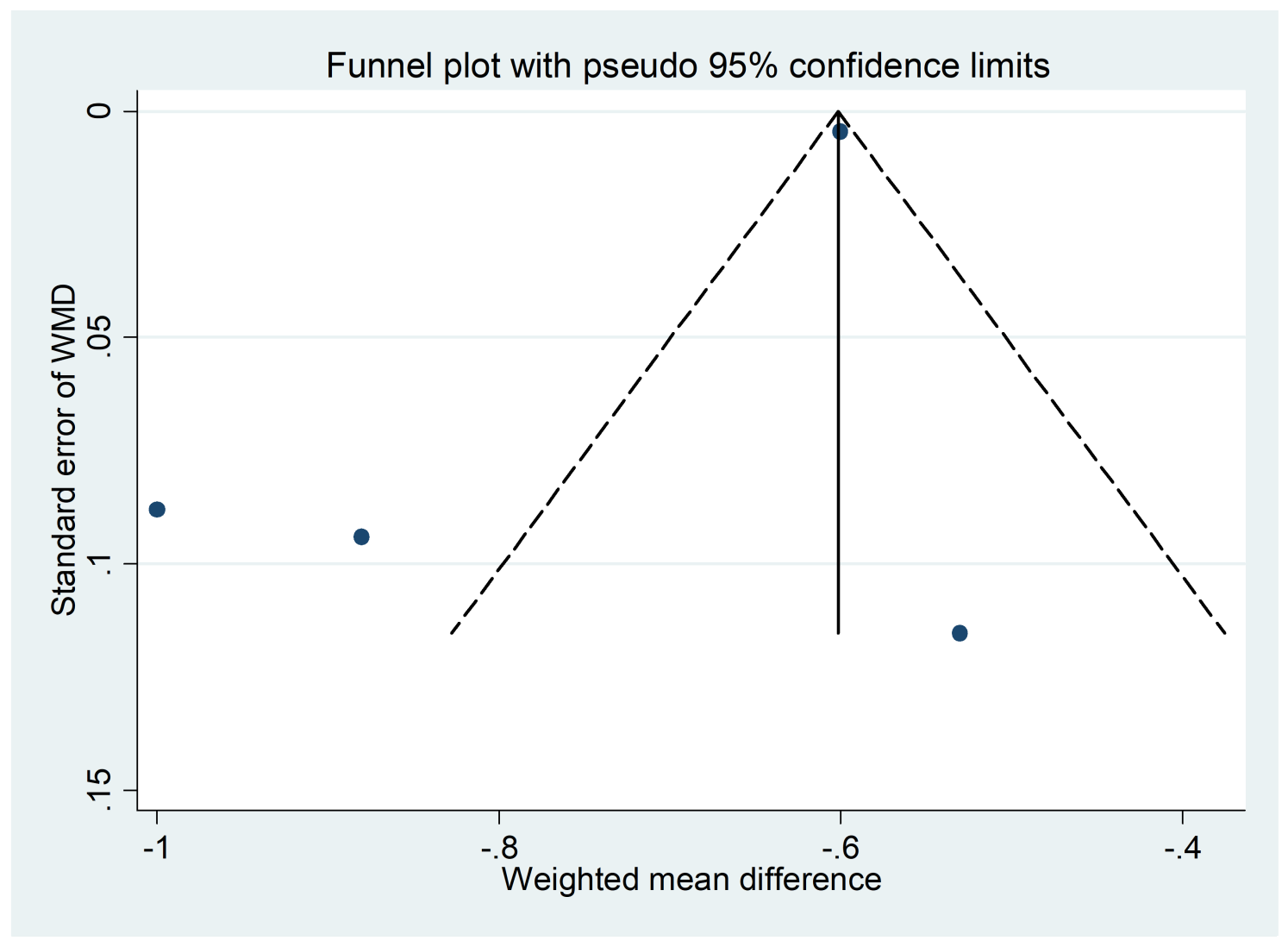
**

**PRISMA 2020 for abstracts checklist**

| **Section and Topic** | **Item #** | **Checklist item** | **Reported (Yes/No)** |
| --- | --- | --- | --- |
| **TITLE** | | |  |
| Title | 1 | Identify the report as a systematic review. | Yes |
| **BACKGROUND** | | |  |
| Objectives | 2 | Provide an explicit statement of the main objective(s) or question(s) the review addresses. | Yes |
| **METHODS** | | |  |
| Eligibility criteria | 3 | Specify the inclusion and exclusion criteria for the review. | Yes |
| Information sources | 4 | Specify the information sources (e.g. databases, registers) used to identify studies and the date when each was last searched. | Yes |
| Risk of bias | 5 | Specify the methods used to assess risk of bias in the included studies. | Yes |
| Synthesis of results | 6 | Specify the methods used to present and synthesise results. | Yes |
| **RESULTS** | | |  |
| Included studies | 7 | Give the total number of included studies and participants and summarise relevant characteristics of studies. | Yes |
| Synthesis of results | 8 | Present results for main outcomes, preferably indicating the number of included studies and participants for each. If meta-analysis was done, report the summary estimate and confidence/credible interval. If comparing groups, indicate the direction of the effect (i.e. which group is favoured). | Yes |
| **DISCUSSION** | | |  |
| Limitations of evidence | 9 | Provide a brief summary of the limitations of the evidence included in the review (e.g. study risk of bias, inconsistency and imprecision). | No |
| Interpretation | 10 | Provide a general interpretation of the results and important implications. | Yes |
| **OTHER** | | |  |
| Funding | 11 | Specify the primary source of funding for the review. | N/A |
| Registration | 12 | Provide the register name and registration number. | N/A |

Abbreviations: PRISMA, Preferred Reporting Items for Systematic Reviews and Meta-Analyses; N/A, not applicable.

**PRISMA 2020 checklist**

| **Section and Topic** | **Item #** | **Checklist item** | **Location where item is reported** |
| --- | --- | --- | --- |
| **TITLE** | | |  |
| Title | 1 | Identify the report as a systematic review. | Page 1 |
| **ABSTRACT** | | |  |
| Abstract | 2 | See the PRISMA 2020 for Abstracts checklist. | Page 2 |
| **INTRODUCTION** | | |  |
| Rationale | 3 | Describe the rationale for the review in the context of existing knowledge. | Page 3 |
| Objectives | 4 | Provide an explicit statement of the objective(s) or question(s) the review addresses. | Page 3 |
| **METHODS** | | |  |
| Eligibility criteria | 5 | Specify the inclusion and exclusion criteria for the review and how studies were grouped for the syntheses. | Page 3 |
| Information sources | 6 | Specify all databases, registers, websites, organisations, reference lists and other sources searched or consulted to identify studies. Specify the date when each source was last searched or consulted. | Page 3 |
| Search strategy | 7 | Present the full search strategies for all databases, registers and websites, including any filters and limits used. | Table S1/2 |
| Selection process | 8 | Specify the methods used to decide whether a study met the inclusion criteria of the review, including how many reviewers screened each record and each report retrieved, whether they worked independently, and if applicable, details of automation tools used in the process. | Page 3 |
| Data collection process | 9 | Specify the methods used to collect data from reports, including how many reviewers collected data from each report, whether they worked independently, any processes for obtaining or confirming data from study investigators, and if applicable, details of automation tools used in the process. | Page 4 |
| Data items | 10a | List and define all outcomes for which data were sought. Specify whether all results that were compatible with each outcome domain in each study were sought (e.g. for all measures, time points, analyses), and if not, the methods used to decide which results to collect. | Page 4 |
|  | 10b | List and define all other variables for which data were sought (e.g. participant and intervention characteristics, funding sources). Describe any assumptions made about any missing or unclear information. | Page 4 |
| Study risk of bias assessment | 11 | Specify the methods used to assess risk of bias in the included studies, including details of the tool(s) used, how many reviewers assessed each study and whether they worked independently, and if applicable, details of automation tools used in the process. | Page 4 |
| Effect measures | 12 | Specify for each outcome the effect measure(s) (e.g. risk ratio, mean difference) used in the synthesis or presentation of results. | Page 4 |
| Synthesis methods | 13a | Describe the processes used to decide which studies were eligible for each synthesis (e.g. tabulating the study intervention characteristics and comparing against the planned groups for each synthesis (item #5)). | Page 3 |
|  | 13b | Describe any methods required to prepare the data for presentation or synthesis, such as handling of missing summary statistics, or data conversions. | Page 4 |
|  | 13c | Describe any methods used to tabulate or visually display results of individual studies and syntheses. | Page 4 |
|  | 13d | Describe any methods used to synthesize results and provide a rationale for the choice(s). If meta-analysis was performed, describe the model(s), method(s) to identify the presence and extent of statistical heterogeneity, and software package(s) used. | Page 4 |
|  | 13e | Describe any methods used to explore possible causes of heterogeneity among study results (e.g. subgroup analysis, meta-regression). | Page 4 |
|  | 13f | Describe any sensitivity analyses conducted to assess robustness of the synthesized results. | N/A |
| Reporting bias assessment | 14 | Describe any methods used to assess risk of bias due to missing results in a synthesis (arising from reporting biases). | Page 4 |
| Certainty assessment | 15 | Describe any methods used to assess certainty (or confidence) in the body of evidence for an outcome. | N/A |
| **RESULTS** | | |  |
| Study selection | 16a | Describe the results of the search and selection process, from the number of records identified in the search to the number of studies included in the review, ideally using a flow diagram. | Page 4 |
|  | 16b | Cite studies that might appear to meet the inclusion criteria, but which were excluded, and explain why they were excluded. | Figure 1 |
| Study characteristics | 17 | Cite each included study and present its characteristics. | Page 4-5 |
| Risk of bias in studies | 18 | Present assessments of risk of bias for each included study. | Figure S1 |
| Results of individual studies | 19 | For all outcomes, present, for each study: (a) summary statistics for each group (where appropriate) and (b) an effect estimate and its precision (e.g. confidence/credible interval), ideally using structured tables or plots. | Figure 2, 3, Table S5 |
| Results of syntheses | 20a | For each synthesis, briefly summarise the characteristics and risk of bias among contributing studies. | Page 4 |
|  | 20b | Present results of all statistical syntheses conducted. If meta-analysis was done, present for each the summary estimate and its precision (e.g. confidence/credible interval) and measures of statistical heterogeneity. If comparing groups, describe the direction of the effect. | Page 5-6 |
|  | 20c | Present results of all investigations of possible causes of heterogeneity among study results. | Figure 2, 3 |
|  | 20d | Present results of all sensitivity analyses conducted to assess the robustness of the synthesized results. | N/A |
| Reporting biases | 21 | Present assessments of risk of bias due to missing results (arising from reporting biases) for each synthesis assessed. | N/A |
| Certainty of evidence | 22 | Present assessments of certainty (or confidence) in the body of evidence for each outcome assessed. | N/A |
| **DISCUSSION** | | |  |
| Discussion | 23a | Provide a general interpretation of the results in the context of other evidence. | Page 6-7 |
|  | 23b | Discuss any limitations of the evidence included in the review. | Page 7 |
|  | 23c | Discuss any limitations of the review processes used. | Page 7 |
|  | 23d | Discuss implications of the results for practice, policy, and future research. | Page 7 |
| **OTHER INFORMATION** | | |  |
| Registration and protocol | 24a | Provide registration information for the review, including register name and registration number, or state that the review was not registered. | Page 9 |
|  | 24b | Indicate where the review protocol can be accessed, or state that a protocol was not prepared. | N/A |
|  | 24c | Describe and explain any amendments to information provided at registration or in the protocol. | Page 9 |
| Support | 25 | Describe sources of financial or non-financial support for the review, and the role of the funders or sponsors in the review. | N/A |
| Competing interests | 26 | Declare any competing interests of review authors. | Page 9 |
| Availability of data, code and other materials | 27 | Report which of the following are publicly available and where they can be found: template data collection forms; data extracted from included studies; data used for all analyses; analytic code; any other materials used in the review. | N/A |

Abbreviations: PRISMA, Preferred Reporting Items for Systematic Reviews and Meta-Analyses; N/A, not applicable.

**PICO protocol**

The PICO question was ‘When a patient with type 2 diabetes mellitus (T2DM) is inadequately controlled using glucagon-like peptide-1 receptor agonists (GLP-1RA), what is the relative effect of free up-titration of added basal insulin compared to fixed-ratio combination (FRC) of basal insulin and GLP-1RA?’

1. Population: Patients with T2DM who were uncontrolled with GLP-1RA.
2. Intervention: Addition of basal insulin with one of two strategies: 1) free up-titration, 2) FRC of insulin and GLP-1RA.
3. Comparison: Maintenance of the previous GLP-1RA.
4. Outcomes: Primary outcome was the change in HbA1c from baseline. Secondary outcomes were the change in fasting plasma glucose levels, the proportion of patients with HbA1c <7.0% at the end of the trial, and the incidence of hypoglycemia.

Eligible articles were randomized controlled trials conducted with patients with T2DM uncontrolled with GLP-1RA at any age and for any sex, comparing the addition and free up-titration of basal insulin or FRC with continuing GLP-1RA, reporting HbA1c change from baseline to the endpoint, and being published in English without publication year restriction.
